# Editorial: Role of the IL-23/IL-17 Pathway in Chronic Immune-Mediated Inflammatory Diseases: Mechanisms and Targeted Therapies

**DOI:** 10.3389/fimmu.2021.770275

**Published:** 2021-09-23

**Authors:** Elisabetta Bianchi, Matteo Vecellio, Lars Rogge

**Affiliations:** ^1^ Immunoregulation Unit, Department of Immunology, Institut Pasteur, Paris, France; ^2^ Unité Mixte de Recherche, Institut Pasteur/AP-HP Hôpital Cochin, Paris, France; ^3^ Nuffield Department of Orthopaedics, Rheumatology and Musculoskeletal Sciences, University of Oxford, Oxford, United Kingdom; ^4^ Division of Rheumatology and Clinical Immunology, Humanitas Clinical and Research Center, IRCCS, Milan, Italy

**Keywords:** chronic inflammatory diseases, spondyloarthritis, interleukin-23, interleukin-17, psoriasis

Chronic inflammatory diseases (CID) are clinically heterogeneous conditions that share common inflammatory pathways and derive from aberrant immune responses. The implication of the interleukin-23/interleukin-17 (IL-23/IL-17) axis in several CID is supported by studies in animal models of autoimmune disease (1, 2) and by the genome-wide association studies (GWAS) finding that several of the non-MHC loci genetically linked to Crohn’s disease, psoriasis, and axial spondyloarthritis (axSpA), are associated with genes in this pathway (*IL23R*, *IL12B*, *IL6R*, *IL1R2*, *RORC*, *RUNX3*, *TYK2*, *JAK2*, *CARD9*) (3–5).

The clinical relevance of the IL-23/IL-17 axis has been validated by the successful treatment of psoriasis, psoriatic arthritis (PsA) and axSpA with IL-17A inhibitors (6–8). Furthermore, targeting IL-23 has proven highly effective for the treatment of psoriasis, and beneficial in PsA (9, 10), a disease belonging to the SpA spectrum. However, the clinical studies using these drugs have also given unexpected results, dissociating the effectiveness of IL-17 from IL-23 inhibitors in different diseases (11). A recent phase 2 study testing the IL-23 inhibitor risankizumab did not show any clinically improvement compared to placebo in patients with active axial SpA (12), despite the strong GWAS association of *IL23R* with SpA (4). Conversely, targeting IL-23 has proven effective for the treatment of Crohn’s disease, while IL-17 inhibition induced worsening of symptoms in this disease (13).

These findings demonstrate our limited understanding of the pathogenic mechanisms of IL-17 and IL-23 in these CIDs, suggesting the need to reassess the link between IL-23 and IL-17 in these diseases.

This Research Topic offers an overview of the impact of the IL-23/IL-17 pathways in CIDs, in particular SpA, with a focus on the mechanisms driving pathogenesis and response to therapy.

IL-23 is important for the expansion and the functional activity of T helper 17 (Th17) cells, which secrete the pro-inflammatory cytokine IL-17 (14), but it may also act on several populations of innate immune cells that express the IL-23 receptor (IL-23R), including innate lymphoid cells (ILC), γδ T lymphocytes, iNKT cells, mucosal-associated invariant T cells (MAIT), and, neutrophils (15–20). Some of these populations have been found to accumulate in the diseased tissues of patients or of model animals (21), suggesting that the inflammatory response in CID may be the result of a complex interplay of different immune cell types whose relative role in the pathogenesis of specific CIDs remains to be defined. Rosine and Miceli-Richard provide a comprehensive overview of IL-17 producing innate cell subsets in the context of SpA pathogenesis, while McGinty et al. propose that the immunoregulatory function of Tr1 cells may be impaired in SpA. IL-10 production by Tr1 was shown to prevent gut inflammation, and IL-23 downregulates IL-10 secretion in these cells (22). Given the therapeutic potential of these cells, the future challenge is the development of appropriate pre-clinical models to explore the role of Tr1 cells in CIDs.

Another cytokine regulated by IL-23 is IL-22, which is produced by Th17 cells, among other cell subsets (23). Lindhal and Olson explore in detail the role of IL-22 in Th1/Th17 cell polarization and in CIDs. Although the different studies are not always consistent, IL-22 seem in most models to reduce Th1 responses and may contribute to resolve inflammation by inducing IL-10 production.

Th17 differentiation is regulated at the transcriptional level by the IRF4 transcription factor (24). Using a T cell transfer model of colitis, Buchele et al. demonstrate that IRF4 also controls Th17 pathogenic function indirectly, by acting in a conventional Dendritic Cell 2 (cDC2) subset. This work highlights the role of IRF4 as a molecular switch that controls Th17 differentiation, as well as the importance of the cDC2 subset in the pathogenesis of colitis.

Adding complexity to the regulation of Th17 function, Peng et al. demonstrate a post-transcriptional mechanism that controls IL-17-mediated inflammation. The authors have shown that Tristetraprolin (TTP), an RNA-binding protein, inhibits IL-23 expression. In the present work, Peng et al. show that TTP conditional KO (CD4CreTTP^f/f^) mice displayed increased systemic IL-17A and skin Th17 cells, and increased susceptibility to DSS-induced colitis. These data indicate that TTP is an important regulator of inflammation and a potential new therapeutic target.

Animal models of CID have been crucial to improve our understanding of the molecular processes that drive CIDs, as comprehensively illustrated by Mandour et al., in particular for diseases such as axSpA, for which access to human diseased tissue is difficult. Rodent models for SpA have been useful to study the molecular mechanisms of IL-23 induced pathogenesis, despite the fact that none reflects the whole range of pathologic findings of this disease. The study of these models has highlighted the possible role of IL-23-dependent gut and skin inflammation in triggering joint pathology. Another interesting finding of these studies is the importance of IL-23 in the early phases of SpA pathogenesis, demonstrated by the ability of IL-23 blockers to prevent disease onset when administered before the development of symptoms. The study of early events in these models may help develop predictive tools and identify targets for early therapeutic intervention. Whether IL-23 plays a similar role in the pre-clinical phase of the disease in humans remains to be established.

In human studies, GWAS have proven very useful to indicate potential pathogenic pathways in CIDs.

Disease-associated genetic variants may have the power to discriminate between similar conditions, such as psoriasis, PsA and ankylosing spondylitis (AS). In their article, Vecellio et al. highlighted the contribution of the IL-17/IL-23 axis to PsA, a disorder sharing most of the genetics and molecular mechanisms with other inflammatory diseases, like psoriasis, AS, inflammatory bowel disease and Behçet disease. The association of loci in the IL-17/IL-23 axis is the *usual suspect* that characterizes these disorders, together with the contribution of Th17 lymphocytes. The development of biologics blocking IL-17, such as Secukinumab in AS, or IL-23 such as Ustekinumab in psoriasis/PsA, demonstrates the value of a combination of genetic markers as an approach to identify credible targets for treatment. Wordsworth et al. elegantly summarize the progress made in the last years by the research community to identify candidate genes that contribute to increased AS susceptibility. More than 100 loci have been found to be associated with increased AS risk, but this could be an underestimate: it is crucial to have large cohorts and bigger sample size to increase the power of these studies. The authors point out that still no reliable genetic predictors of disease severity in AS or response to treatment are available, despite the efforts of the scientific community. The identification of credible therapeutic targets and the translation of genome wide association studies (GWAS) findings in AS, is the main massage from Zaroour et al.’s contribution. Despite recent progress, several challenges are still present in order to predict which are the causal genes regulated by disease-associated genetic variants and to define the relevant cell-type where these SNPs act. The success of biologics targeting the IL-17/IL-23 axis highlights the value of genetic studies for drug development. However, since it’s very unlikely that two patients will have the same genetic makeup, stratification based on genetic predictors remains challenging.

Schinocca et al. summarize recent findings in human and animal models supporting the role of the IL-23/IL-17 pathway in SpA and other rheumatic diseases, including Rheumatoid Arthritis, Sjögren Syndrome and Systemic Lupus Erythematosus, linking molecular pathology to the development of biologic therapies. Several novel biologics targeting the IL-23/IL-17 pathway (including IL-17F and the Janus kinases (JAK) downstream of IL-23R) are being currently tested, as detailed by Ceribelli et al. in their comprehensive review of ongoing clinical trials for SpA treatment.

Hammitzsch et al. focus their attention on the role of JAKs in SpA pathogenesis, and the development of inhibitors for treatment. Given the pleiotropic role of a JAK in multiple signaling pathways, they argue for the use of selective inhibitors, to avoid undesired alterations of bone homeostasis.

In a bedside to bench approach, Fiechter et al. interrogate the effect of IL-23 inhibition on molecular pathways and cellular populations in the synovia of PsA patients. Major pathways modulated by treatment were MAPK/ERK, mTOR and Wnt signaling, while IL-17A production was not significantly affected, supporting a non-linearity in the IL-23/IL-17 pathway, and the possibility of other pathogenic targets downstream of IL-23. Changes induced by treatment in the Wnt pathway also indicate the importance of better investigating the effects of IL-23 on bone metabolism.

Liu et al. explore the role of IL-23 and the effects of its inhibition in a wide range of inflammatory skin diseases. Their analysis suggests that IL-23 may be important for the development of several skin diseases, including Hidradenitis Suppurativa or Pityriasis Rubra, which show clinical improvement upon IL-23 blockade. Bugaut and Aractingi focus on the pathogenesis and treatment of psoriasis, with an eye to new therapeutics inhibiting selective JAKs and the transcription factor RORγt, which is essential for the function of Th17 cells. The authors underline how improved understanding of IL-23/IL-17 biology, and of the many cell types involved, may lead to the identification of new therapeutic targets, necessary for severe and refractory cases.

The role of the IL-23/IL-17 axis in inflammatory bowel disease (IBD) is reviewed by Schmitt et al. and by Noviello et al. An interesting concept that emerges from this overview is the importance of considering T cell plasticity and changes in immune profiles during disease progression, which may explain the need for a different biological treatment at different stages of disease. Noviello et al. also suggest the possibility of stratifying patients for treatment according to baseline cytokine levels.

Finally, Baeten and Adamopoulos and McGonagle et al. discuss potentials reasons why IL-23-inhibition failed in AS. Revisiting the scientific rationale for conducting trials of IL-23-inhibitors in AS, Baeten and Adamopoulos caution that the evidence supporting a central role of IL-23 in the pathobiology of AxSpA was circumstantial, at best. In particular, they state that systemic IL-23 exposure induced chronic arthritis, severe bone loss and myelopoiesis in the bone marrow and spleen of mice (25), a phenotype which is not compatible with AxSpA. This report clearly contrasts the more publicized observation that IL-23 overexpression induces a SpA-like phenotype in mice (26). This perspective concludes with the notion that the IL-23/IL-17 axis is not a linear “cascade” and that genetic data are an excellent tool to generate hypotheses, but are not sufficient to prove or disprove them. McGonagle et al. argue that IL-23 blockade can prevent disease onset but not established disease in an experimental SpA model (27). Even if it is currently not clear if these observations can be translated to human disease, they point to a role of IL-23 in disease initiation, while persistent disease may be maintained by IL-23-independent IL-17 production by memory T cells. IL-23-independent IL-17 production by various lymphocyte populations is discussed in several reviews in this topic. McGonagle et al. point out that there is heterogeneity within human γδ T cell populations with respect to IL-23 receptor expression. Both δ1 and δ2 γδ T cell populations express IL-17A following stimulation, however only the δ2 population further upregulated IL-17A production when stimulated in the presence of IL-23 (16). An interesting point also discussed by McGonagle et al. is that patients with active PsA and imaging-confirmed sacroiliitis (axial PsA) benefit from treatment with the anti-IL-12/23 inhibitor ustekinumb (28) and the IL-23 inhibitor guselkumab (29). Thus, a subgroup of SpA patients with axial inflammation may actually benefit from IL-23-blockade, and McGonagle et al. propose that adequate IL-23 posology may be critical in this condition.

In conclusion, the articles included in this collection reached their “primary endpoint”, that is raising more questions to guide future clinical and fundamental research.

## Author Contributions

All authors listed have made a substantial, direct, and intellectual contribution to the work and approved it for publication.

## Conflict of Interest

The authors declare that the research was conducted in the absence of any commercial or financial relationships that could be construed as a potential conflict of interest.

## Publisher’s Note

All claims expressed in this article are solely those of the authors and do not necessarily represent those of their affiliated organizations, or those of the publisher, the editors and the reviewers. Any product that may be evaluated in this article, or claim that may be made by its manufacturer, is not guaranteed or endorsed by the publisher.
